# 20-Year labor market histories of 66-year-old women and men: a nationwide retrospective cohort study from Sweden

**DOI:** 10.1093/eurpub/ckag062

**Published:** 2026-04-16

**Authors:** Katalin Gémes, Aleksiina Martikainen, Kristin Farrants, Kristina Alexanderson, Jakob Bergström, Ellenor Mittendorfer-Rutz, Marianna Virtanen

**Affiliations:** Department of Clinical Neuroscience, Karolinska Institutet, Stockholm, Sweden; Department of Clinical Neuroscience, Karolinska Institutet, Stockholm, Sweden; Department of Clinical Neuroscience, Karolinska Institutet, Stockholm, Sweden; Department of Clinical Neuroscience, Karolinska Institutet, Stockholm, Sweden; Department of Clinical Neuroscience, Karolinska Institutet, Stockholm, Sweden; Department of Clinical Neuroscience, Karolinska Institutet, Stockholm, Sweden; Department of Clinical Neuroscience, Karolinska Institutet, Stockholm, Sweden; School of Educational Sciences and Psychology, University of Eastern Finland, Joensuu, Finland

## Abstract

A health-promoting, supportive, and inclusive labor market is essential to sustainable working life. However, knowledge is warranted on working life patterns from midlife on. We aimed to map 20-year histories of labor market and healthcare use among women and men aged 66. A 20-year retrospective cohort study of 52 920 women and 51 823 men aged 66 in 2019 who lived in Sweden 2000–19, using microdata on type of economic activity, income, secondary healthcare use, and prescribed medications, linked from nationwide registers. Sequence and cluster analysis were performed on yearly dominant labor market states, separately for women and men. Women spent more time in “low-income” and “sickness absence/disability pension (SA/DP)” and less time in “high-income” states than men. Time spent in “unemployed,” “no/minimal income,” “social assistance” and “retired” states were similar in both sexes. Probabilities of transitioning from “SA/DP,” “social assistance,” and “retired” to other states were low (<.09). The largest sequence cluster in men (68%) was mostly characterized by sequences with “high-income” and in women by “low-” and “high-” income states (74%). Other clusters were represented by “unemployed” (14% of women, 17% of men), “no/minimal income” (10%, 11%), and “SA/DP” (3%, 3%) states. Most women and men were active in the labor market when aged 46–66, nevertheless, around 30% followed less active paths, dominated by long-term SA/DP, social assistance, or unemployment. Transitions from these states were unlikely. The most pronounced sex-difference in working life was time spent in “low”- and “high-income” states.

## Introduction

Many European countries are preparing for the impact that the aging populations will have on labor market, healthcare, and welfare systems [1], e.g., by investigating possible reforms to the public pension systems to support extended working lives. In Sweden, the societal normative retirement age was 65 years for decades, and several government policies have recently been implemented with the aim to increase the age of retirement and provide incentives for people to prolong their working lives [2]. To extend working life and maintain work capacity at higher ages, it is crucial to promote good health and ensure sustainable and inclusive participation in the labor market throughout a person’s working life [2].

A major concern for achieving a just and sustainable working life is the difference between women and men; in European countries, women have lower labor market participation throughout working life and exit the workforce earlier than men [2, 3]. This can be related to various reasons, including time spent in unpaid work (e.g., caring for children or other family members), higher morbidity during working age (e.g., breast cancer, autoimmune disorders, and common mental disorders) [2–4], and temporary or part-time jobs, often characterized by instability and insecurity [2, 5, 6]. Moreover, the labor market is sex segregated [7, 8], and numerically female-dominated occupations tend to have higher psychosocial workload [5, 6, 8], poorer control over working hours [9], and higher rates of workplace bullying [10]. Men, in turn, are more likely to work longer hours, report higher effort-reward imbalance, lower social support, and higher physical job demands [9].

Many morbidities and work characteristics are associated with interruptions in working-life participation or early labor market exit in terms of long-term sickness absence (SA), disability pension (DP) [4, 11, 12], and early retirement [13, 14]. Few studies have investigated labor market patterns before retirement from an integrative life-course perspective [15], which includes both health- and work-related aspects as reasons for work-life interruption in later working life [14]. Furthermore, previous studies have often relied on survey samples without morbidity data. Therefore, the aim of this study was to map labor market and healthcare use histories among a cohort of women and men of the same age who recently passed the typical retirement age (65) and to identify working life patterns in their 20-year histories. We explored the following research questions: (i) What kind of work and health-related patterns did women and men aged 66 have in the past 20 years? (ii) Were there specific characteristics in those patterns that differed between women and men, such as income and health?

## Methods

A population-based, retrospective cohort study was conducted, based on microdata linked from five Swedish nationwide registers.

### Study population

We included all women and men who lived in Sweden throughout the 20-year period 2000–19 and turned 66 years in 2019, identified from Statistics Sweden’s Longitudinal Integrated Database for Labor Market Studies. Information about the cohort members was linked at the individual level to information from the following nationwide registers: National Patient Register, the Prescribed Drug Register, the Cause of Death Register, all kept by the Board of Health and Welfare, and the Micro-Data for Analysis of the Social Insurance System kept by the Social Insurance Agency. A detailed description of the registers and the Swedish social insurance context is provided in the Supplementary Material.

### Definition of yearly labor market states

The following, mutually exclusive, yearly labor market states were defined based on annual information on net sickness absence/disability pension (SA/DP) days, days on unemployment benefit, income from work, parental leave benefits, student benefits, social assistance, and old-age pension by using a state definition algorithm (Supplementary Fig. S1):

*High-income*: income from work and/or student benefits and/or parental leave benefit above the median income in Sweden (obtained from Statistics Sweden) for the specific year (Supplementary Table S1).

*Low-income*: income that was at least two times the yearly price base amount (“prisbasbelopp” by Statistic Sweden), but less than or equal to the yearly median.

*No/minimal income*: income less than or equal to two times the yearly price base amount without social assistance.

*Unemployed*: unemployment benefits ≥183 days.

*On social assistance*: more than 50% of the individual’s annual income came from social assistance.

*SA/DP*: sum of the SA/DP net days ≥183.

*Retired*: income from old-age pension and no work income above two times the price base amount.

People in all these states could also have capital or undeclared income or be financially supported by others.

### Covariates

Information on birth country, educational level, type of living area, and family situation was obtained for the year 2019. Mental and somatic morbidities were identified based on secondary healthcare use (i.e. specialized outpatient and inpatient healthcare) and prescribed dispensed medication during the observation period (Supplementary Material; Definition of covariates).

### Statistical analysis

All analyses were conducted separately for women and men. We used sequence analysis to examine 20-year labor market histories by identifying temporal sequences of annual labor market activity states [16]. We created sequence index plots, state proportion plots, and a list of the 10 most common sequences and calculated the mean duration of years within a given labor market state.

Similar sequences were grouped into distinct *clusters* using cluster analysis. We calculated dissimilarity measures between the individual sequences using optimal matching spell (OMspell) algorithm with zero expansion cost to consider the order of transitions between the various work and nonwork-related states before age 67 [16, 17]. Emphasis was placed on sequencing rather than duration or timing because the order of labor market states prior to retirement reflects whether and how labor market attachment is maintained and from which state individuals retire. Substitution costs were defined by the frequency of state occupation, i.e. rarely visited states contributed to a higher cost and states visited often yielded a lower cost to emphasize uncommon labor market transitions (Supplementary Table S2a and b). The partition around medoids (PAM) algorithm was used to group similar sequences into clusters [17], and cluster solutions were chosen for further analysis based on several partition quality measures (Supplementary Table S3a and b), membership sizes, and the interpretability of the specific clusters. We named the clusters based on the dominant sequence types, then created the different sequence illustrations, and presented the distribution of predictors by the defined clusters. Data management was performed with SAS (v9.4) and statistical analysis with R (v4.5.2) using TraMineR [18] and WeightedCluster [19] packages. The study was approved by the Regional Ethical Review Board, Karolinska Institutet, Stockholm, and by the Swedish Ethical Review Authority (Dnr 2007/762-31, Dnr2021-06441-02).

## Results

Descriptive statistics of the study variables are presented in Table 1 (women) and Table 2 (men). Most women (85%) and men (87%) were born in Sweden, 36% of women and 31% of men had a college/university education, and 53% of women and 38% of men had a history of any mental and/or somatic morbidity during the observational period.

**Table 1.** Distribution of study variables among women in total and by cluster memberships.

Variable	Total (*N = *52 920)	Mixed income (*N = *39 126)	Low income and unemployment (*N = *7254)	Low/minimal income (*N = *5172)	Sickness and social benefits (*N = *1368)
Type of living area, *n* (%)					
City	18 073 (34.2%)	13 403 (34.3%)	2192 (30.2%)	1750 (33.8%)	728 (53.2%)
Town and suburb	22 269 (42.1%)	16 536 (42.3%)	3201 (44.1%)	2094 (40.5%)	438 (32.0%)
Rural area	12 578 (23.8%)	9187 (23.5%)	1861 (25.7%)	1328 (25.7%)	202 (14.8%)
Educational level, *n* (%)					
Elementary	8682 (16.4%)	5549 (14.2%)	1516 (20.9%)	1009 (19.5%)	608 (44.4%)
High school	24 956 (47.2%)	18 017 (46.0%)	3954 (54.5%)	2444 (47.3%)	541 (39.5%)
University/college	19 282 (36.4%)	15 560 (39.8%)	1784 (24.6%)	1719 (33.2%)	219 (16.0%)
Country of birth, *n* (%)					
Sweden	45 205 (85.4%)	34 608 (88.5%)	5970 (82.3%)	4117 (79.6%)	510 (37.3%)
Nordic (except Sweden)	2720 (5.1%)	1960 (5.0%)	418 (5.8%)	270 (5.2%)	72 (5.3%)
EU (except Denmark, Finland, and Sweden)	1704 (3.2%)	1022 (2.6%)	314 (4.3%)	285 (5.5%)	83 (6.1%)
The rest of the world	3291 (6.2%)	1536 (3.9%)	552 (7.6%)	500 (9.7%)	703 (51.4%)
Family situation, *n* (%)					
Married or cohabitant	30 673 (58.0%)	23 601 (60.3%)	3740 (51.6%)	3033 (58.7%)	299 (21.9%)
Single	22 247 (42.0%)	15 525 (39.7%)	3514 (48.4%)	2139 (41.4%)	1069 (78.1%)
History of mental morbidities,^a^*n* (%)	28 236 (53.4%)	19 990 (51.1%)	4441 (61.2%)	2757 (53.3%)	1048 (76.6%)
History of somatic morbidities,^b^*n* (%)	35 165 (66.4%)	25 860 (66.1%)	4984 (68.7%)	3305 (63.9%)	1016 (74.3%)
History of mental and/or somatic morbidities, *n* (%)	42 275 (79.9%)	30 976 (79.2%)	6061 (83.6%)	4016 (77.6%)	1222 (89.3%)
History of mental and somatic morbidities, *n* (%)	21 126 (39.9%)	14 874 (38.0%)	3364 (46.4%)	2046 (39.6%)	842 (61.5%)

a History of mental disorders based on the following diagnoses codes according to the 10th version of the International Classification of Diseases: F00-F03, F20-29, F30-F33, F40-43, Z73.0, G30-G32 and Anatomical-Therapeutic-Chemical (ATC) classification codes of N05A, N05B, N05C, N06A, N06B, N06C, N07B, having at least two dispensations within a year.

b History of common somatic disorders based on the following diagnoses codes according to the 10th version of the International Classification of Diseases: C00-C97, E10-E14, I00-I99, J45, J46, M00-M99 and Anatomical-Therapeutic-Chemical (ATC) classification codes of A10 having at least two dispensations within a year.

**Table 2.** Distribution of study variables among men in total and by cluster memberships.

Variable	Total (*N = *51 823)	High income (*N = *35 293)	Low income and unemployment (*N = *8989)	Low/minimal income (*N = *5822)	Sickness and social benefits (*N = *1719)
Type of living area, *n* (%)					
Cities	16 948 (32.7%)	11 423 (32.4%)	2691 (29.9%)	1961 (33.7%)	873 (50.8%)
Towns and suburbs	21 990 (42.4%)	15 178 (43.0%)	3949 (43.9%)	2270 (39.0%)	593 (34.5%)
Rural areas	12 885 (24.9%)	8692 (24.6%)	2349 (26.1%)	1591 (27.3%)	253 (14.7%)
Educational level, *n* (%)					
Elementary	12 768 (24.6%)	8379 (23.7%)	2332 (25.9%)	1461 (25.1%)	596 (34.7%)
High school	22 837 (44.1%)	14 895 (42.2%)	4486 (49.9%)	2661 (45.7%)	795 (46.2%)
University/college	16 218 (31.3%)	12 019 (34.1%)	2171 (24.2%)	1700 (29.2%)	328 (19.1%)
Country of birth, *n* (%)					
Sweden	45 255 (87.3%)	32 052 (90.8%)	7601 (84.6%)	4732 (81.3%)	870 (50.6%)
Nordic (except Sweden)	1841 (3.6%)	1155 (3.3%)	352 (3.9%)	249 (4.3%)	85 (4.9%)
EU (except Denmark, Finland, and Sweden)	1202 (2.3%)	679 (1.9%)	233 (2.6%)	216 (3.7%)	74 (4.3%)
The rest of the world	3525 (6.8%)	1407 (4.0%)	803 (8.9%)	625 (10.7%)	690 (40.1%)
Family situation, *n* (%)					
Married or cohabitant	31 727 (61.2%)	23 234 (65.8%)	4912 (54.6%)	3149 (53.9%)	432 (25.1%)
Single	20 096 (38.8%)	12 059 (34.2%)	4077 (45.4%)	2673 (45.9%)	1287 (74.9%)
History of mental morbidities,^a^*n* (%)	19 650 (37.9%)	12 227 (34.6%)	3976 (44.2%)	2327 (40.0%)	1120 (65.2%)
History of somatic morbidities,^b^*n* (%)	34 404 (66.4%)	23 009 (65.2%)	6315 (70.3%)	3849 (66.1%)	1231 (71.6%)
History of mental and/or somatic morbidities, *n* (%)	39 015 (75.3%)	26 005 (73.7%)	7152 (79.6%)	4390 (75.4%)	1468 (85.4%)
History of mental and somatic morbidities, *n* (%)	15 039 (29.0%)	9231 (26.2%)	3139 (34.9%)	1786 (30.7%)	883 (51.4%)

a History of mental disorders based on the following diagnoses codes according to the 10th version of the International Classification of Diseases: F00-F03, F20-29, F30-F33, F40-43, Z73.0, G30-G32 and Anatomical-Therapeutic-Chemical (ATC) classification codes of N05A, N05B, N05C, N06A, N06B, N06C, N07B, having at least two dispensations within a year.

b History of common somatic disorders based on the following diagnoses codes according to the 10th version of the International Classification of Diseases: C00-C97, E10-E14, I00-I99, J45, J46, M00-M99 and Anatomical-Therapeutic-Chemical (ATC) classification codes of A10 having at least two dispensations within a year.

### Sequence analysis

Among the 52 920 women and 51 823 men, we identified 22 978 and 24 698 unique sequences, respectively (Supplementary Figs S2 and S3). The most frequent state at age 47 was “low-income” among women (53.8%) and “high-income” among men (54.6%). At age 66, the “retired” state was the most common in both women (79.6%) and men (76.3%). The 10 most common sequences in women included consecutive “high-income,” consecutive “low-income,” and consecutive “SA/DP” states ending in “retired” state, and consecutive “high-income” sequences. In men, these patterns were similar except that the consecutive “low-income” sequence was not among them (Supplementary Fig. S2). On average, women spent most time in “low-income,” whereas men spent most time in “high-income” state (Supplementary Fig. S4). The mean time spent on “SA/DP” was higher among women than men, while the mean time spent in other states (“no/minimal income,” “unemployed,” “on social assistance,” and “retired”) was similar among women and men (Supplementary Fig. S4). Remaining in the same state was substantially more probable than transitioning between states (Supplementary Table S3a and b). The highest probability for different-state transition was the transition from “unemployed” to “low-income” among women (0.17) and from “no/minimal income” to “low-income” among men (0.17).

### Cluster analysis

#### Identified clusters and their main attributes

**Figure 1.**
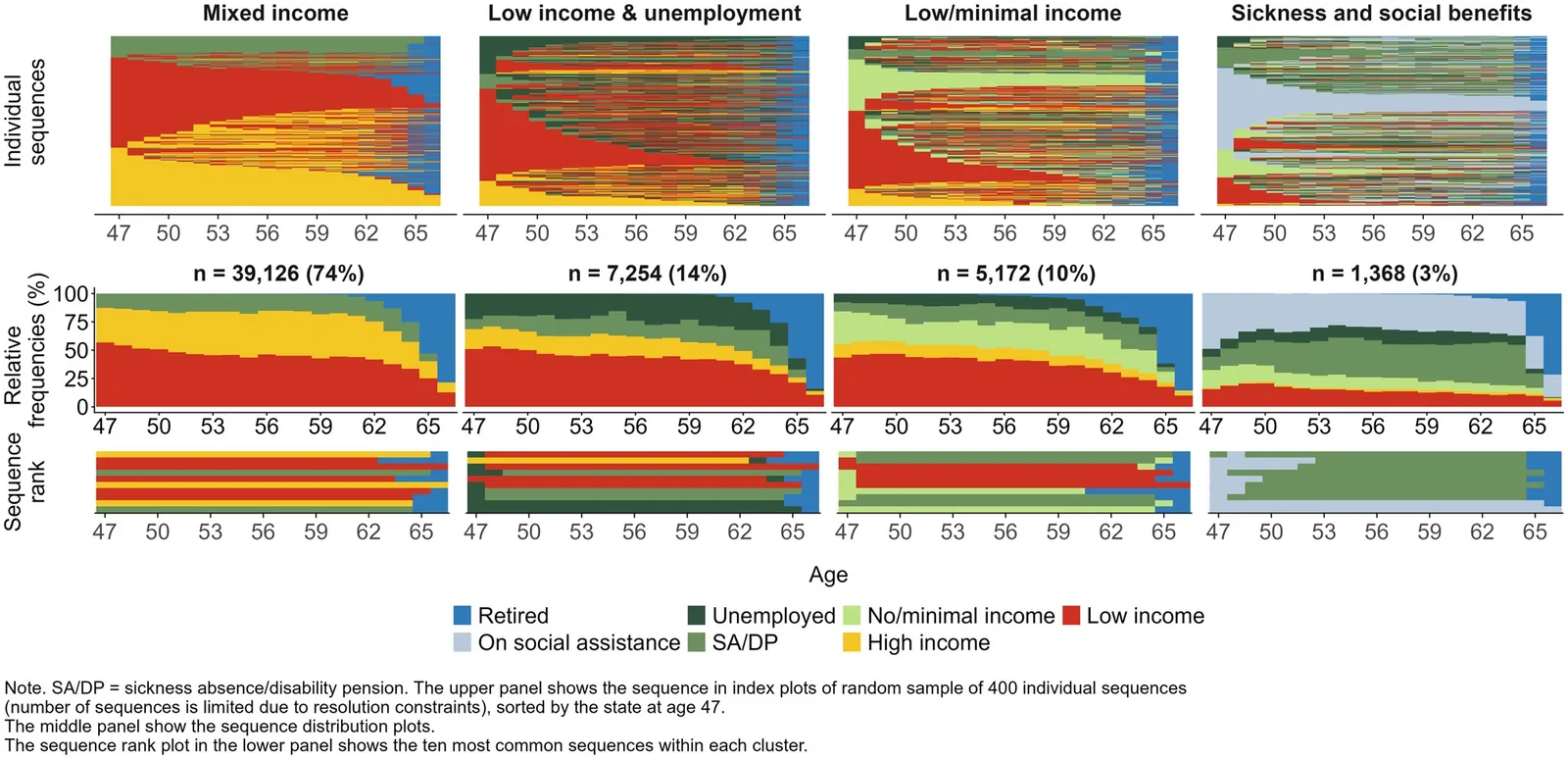
20-Year retrospective labor market sequences by cluster in women aged 66 years in 2019.

**Figure 2.**
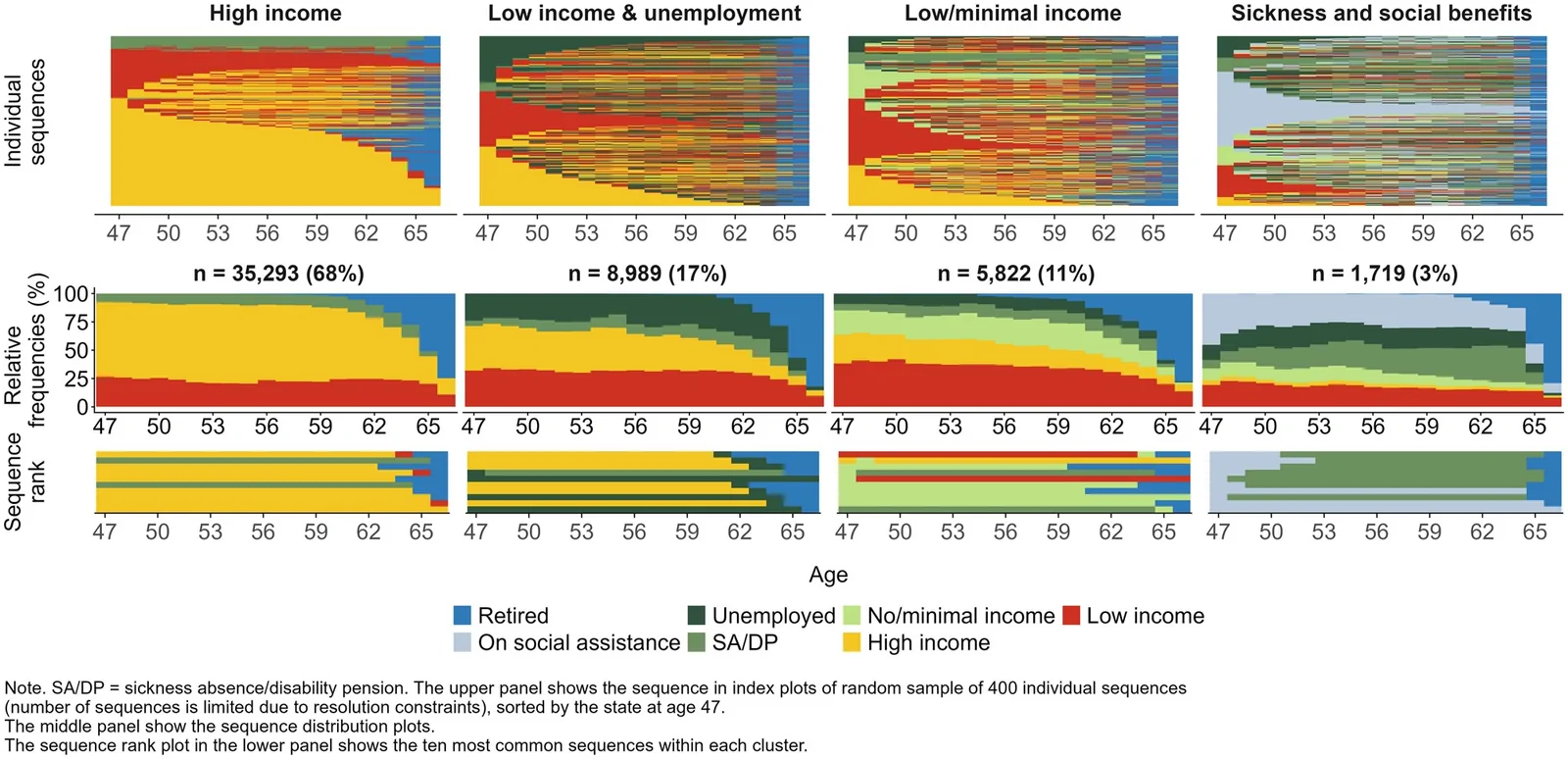
20-Year retrospective labor market sequences by cluster in men aged 66 years in 2019.

Based on the quality measures and the data context, a four-cluster solution was selected both for women and men (Average Silhouette Width, ASW = 0.6 and 0.5, respectively) (Supplementary Table S3a and b, Figs 1 and 2, Supplementary Figs S5a and b and S6a and b). We also present the two-cluster solutions for women (Supplementary Fig. S7a and b) and the three-cluster solution for men (Supplementary Fig. S8a and b) for which the ASW values were similarly high, however, we ultimately chose the four-cluster solutions to better capture important complexity. The clusters were named based on the most representative sequences. Below, the characteristics of each cluster are described among the women and men, respectively.

##### **Women**

Among women, the following four clusters were identified (Fig. 1, Supplementary Figs S5a and 6a):

1. *Mixed income* (*n* = 39 126; 74% of all women)
  This was the largest cluster among women. These women’s labor market sequences were characterized by paid work with varying income levels and a relatively small, stable proportion on “SA/DP” throughout ages 47–66. A gradual transition into the “retired” state began around age 62–63.
2. *Low-income and unemployment* (*n* = 7254; 14%)
  This cluster was marked by “unemployed” state, which was especially common at the beginning of follow-up (at age 47) and relatively stable across the follow-up. “Low-income” state was also relatively common, but fewer women had sequences that included “high-income” state. As in the *Mixed income* cluster, a similar gradual shift into “retired” state was observed.
3. *Low-minimal income* (*n* = 5172; 10%)
  This cluster was characterized by persistent periods in “low-income” and “no/minimal income” states, together with sequences dominated by “low-income” state. The transition to “retired” state happened somewhat earlier compared to other clusters.
4. *Sickness and social benefits* (*n* = 1368; 3%)
  This cluster stood out due to a high proportion of women on “SA/DP” or “on social assistance” state, and a low proportion in paid work, typically in “low-income” state. Compared with other clusters among women, the transition to “retired” state occurred later and more abruptly at age 65, possibly reflecting long periods in “SA/DP” state before retirement age and thereafter transition to old-age retirement.

##### **Men**

Among men, the following four clusters were identified (Fig. 2, Supplementary Figs S5b and S6b):

1. *High-income* (*n* = 35 293; 68% of all men)
  This was the largest cluster among men. These men’s labor market sequences were characterized by paid work with “high-income” across most of the follow-up and a small and stable proportion in “SA/DP” state. Most men remained in “high-income” state until ages 62–63, where after a gradual transition to “retired” state was observed.
2. *Low-income and unemployment* (*n* = 8989; 17%)
  This cluster was marked by a relatively stable proportion of men in “unemployed” state, combined with sequences of paid work in both “low-income” and “high-income” states. The transition to “retired” state followed a similar gradual pattern as in the *High-income* cluster.
3. *Low/minimal income* (*n* = 5822; 11%)
  This cluster stood out with persistent periods of “low-income” and “no/minimal income” states, together with sequences dominated by “low-income” state. The transition to “retired” state occurred somewhat earlier than in the other clusters.
4. *Sickness and social benefits* (*n* = 1719; 3%)
  This cluster was characterized by a high proportion of men in “SA/DP” or “on social assistance” state, and a low proportion of men in paid work, typically in “low-income” state. Among women in the corresponding cluster, the transition to “retired” state occurred sharply at age 65, likely reflecting the upper age limit for DP in Sweden.

#### Differences between clusters

Tables 1 and 2 show that the distribution of sociodemographic and morbidity-related variables varied across clusters for women and men. The differences between clusters were similar among women and men, but were generally more pronounced among women. The largest differences were observed between those engaged in paid work without a history of unemployment (the *Mixed-income* cluster among women and the *High-income* cluster among men) and those in the *Sickness and social benefits* cluster. For example, the proportion with university/college was higher in the *Mixed-income* and *High-income* clusters (women: 40%; men: 34%) than in the *Sickness and social benefits* cluster (women: 16%; men: 19%). A similar pattern was seen for country of birth, with a higher proportion of individuals born in Sweden in the *Mixed-income* and *High-income* clusters (women: 89%; men: 91%) than in the *Sickness and social benefits* cluster (women: 37%; men: 51%). The proportion of individuals with a history of mental and/or somatic morbidity was also highest in the *Sickness and social benefits* cluster (women: 89%; men: 85%).

## Discussion

In this large population-based retrospective cohort study, we mapped 20 years of labor market histories among the 66-year-old women (*n = *52 920) and men (*n = *51 823) in Sweden and identified sociodemographic and health-related factors associated with these patterns. In summary, both women and men followed a range of working life trajectories across ages 47–66. The most common sequences involved transitions from “low-income” and “high-income” states into “retired” state. More men remained in stable sequences of “high-income” state, while women experienced periods of “low-income” and sickness absence and/or disability pension (“SA/DP”) more frequently than men. Four distinct clusters were identified among women and men, respectively. The distribution of sociodemographic and health-related characteristics varied across clusters, but the patterns were similar in both sexes: a higher proportion of individuals in the *SA/DP* clusters had lower education, were born outside the EU, were single, and had a history of mental and/or somatic morbidity.

### Comparison with previous studies

We found no major sex difference in the average time spent in the “retired” state; neither representative sequences nor clusters revealed any substantial differences between women and men in time spent in the “retired” state. Approximately 20% of 66-year-old women and men, respectively, were in the “low-income” or “high-income” states at the end of follow-up in 2019, indicating that they were in paid work, which is in line with what has been reported from other sources [20, 21]. The results from previous research have focused on early retirement which is a different outcome than timing of old-age pension or working at age 66 in our study [22–25]. One explanation for the time spent in the “retired” state in women and men could be that Sweden, like other Scandinavian countries, actively supports the dual-earner model. This model includes comprehensive publicly funded caregiving services with the goal of a more equal contribution of income and care-work in the family and focuses on employment maintenance at older ages [26–28], supported by individual-based taxation.

Nevertheless, we found clear sex difference in income levels across the 20-year labor market histories. More specifically, women’s working life histories were more likely to be characterized by a mix of “low-income,” and “high-income” and predominantly “low-income” states, while men’s histories were more likely to be characterized by “high-income” state. It is also noteworthy that very few appeared to have improvements in their labor market situation, as the probability of transitioning from “SA/DP” or from “on social assistance” to “low-income” and to “high-income” states was low. A history of “low-income” state among women is in line with previous findings, indicating an income gap between women and men in Sweden, as in other countries [27, 28]. Several factors could explain the income difference between women and men. For example, women are more likely to work part-time [28] and engage in unpaid work (such as domestic work and caregiving of partner, relative, or child) than men [29], which might result in a lower average work income. Furthermore, a higher proportion of women than men work in the public sector and/or in occupations within social-, healthcare, and education, where the average salary is lower compared to that in the private sector and other occupational branches. [30] Moreover, women have a higher average rate of SA and DP days than men [31, 32], which might contribute to the observed lower work income [33]. We also found some indication of more SA/DP days among women, reflected by the higher mean time spent in the “SA/DP” state. Reasons behind higher SA rates in women are multifaceted and not fully understood. Some of the differences can be related to that working-aged women have a higher prevalence of morbidity than men [34]. However, most people with morbidity are not sickness absent [35], and associations between morbidity and SA/DP have not been studied much. Furthermore, jobs in numerically female-dominated occupations and sectors often have high physical and psychosocial workload and less control over working time, which might explain some of the higher SA/DP [7–9].

Although there have been several retirement reforms and initiatives to extend working life by creating sustainable working-life policies and practices, previous studies show that health problems remain as one of the main barriers to extending working life [3]. A systematic review found that better health is correlated with working after normative retirement age [36]. Our results align with this pattern, as the probability of returning to paid work after the “SA/DP” state was almost zero, and in this age group, people were most likely to transfer to the “retired” state. Other non-work states (“unemployed”, “on social assistance”, and “no/minimal income”) were also stable, and were often followed by the “retired” state. This may suggest that policies aimed at extending working life could risk increasing employment and income inequalities, as individuals already detached from the labor market may be less likely to return to paid work and instead remain in non-active states such as “SA/DP” or other social benefits before transitioning to retirement. More studies about this topic are warranted. Furthermore, other factors, such as inflexible work, age-, and sex-discrimination might also contribute to early retirement and increase inequalities [37].

The sociodemographic characteristics and morbidities were similar for women and men within the identified working life patterns. These findings are in line with a prior study of individuals aged 16–64, which described that lower education, being born outside the EU, living in rural areas, and having a history of mental disorders were associated with a higher likelihood of labor market marginalization in terms of long-term unemployment and SA/DP [38]. These sociodemographic and morbidity-related factors might contribute to barriers to extending working life.

### Strengths and limitations

A major strength of this study is the population-based cohort design, which includes an entire age cohort and the use of individual-level linked register information with high validity. This enabled us to capture the diversity of 20-year previous labor market patterns and the sociodemographic characteristics and morbidity associated with these labor market histories. The assessment of labor market history, as well as other factors, was based on information from administrative registers, which minimizes misclassification due to self-report and missing information due to non-response.

Our study has some limitations. First, we only included 66-year-old individuals who were living in Sweden in the whole period 2000–19, which limits our generalizability to individuals who moved in or out of Sweden or died during this period. However, outmigration is low in this age group. Second, due to the structure of the data, we could only define labor market states on a yearly basis, which limited our possibility to capture detailed diversities in labor market patterns. Furthermore, information from the Prescribed Drug Register was only available from July 2005, therefore, we only partly reconstructed medication history in the case of mental disorders and diabetes. However, most of the included diagnoses have a chronic nature. Therefore, prevalent cases were likely captured by using later information on prescribed medication, and more severe cases treated in secondary healthcare could be identified throughout the whole observation period. Furthermore, unmeasured occupational exposures might also have contributed to the observed working life patterns.

## Conclusions

Our population-based longitudinal register study shows that women and men had somewhat diverse working life patterns between ages 47 and 66. Most women and men were in paid work throughout the observation period, but the likelihood of transitioning from long-term unemployment, minimal income, and long-term sickness absence (SA/DP) back to active labor market states was minimal. There was no substantial difference between women and men in time spent in retirement before age 66. Women’s working life histories were more likely to include years with low income and long-term SA/DP than men’s, who in turn were more likely to have working life patterns with high income. The findings indicate both economic and health-related sex differences in working life during the last 20 years before retirement age. Further research is needed to identify facilitators and barriers to sustainable working life in women and men. Policy-level actions are needed to focus on reducing inequalities in older age caused by early marginalization and low-income work.

## Supplementary Material

ckag062_Supplementary_Data
