# Supplementary material for: 20-Year labor market histories of 66-year-old women and men: a nationwide retrospective cohort study from Sweden
Source: Eur J Public Health. 2026 Apr 16;36(3):ckag062. doi: 10.1093/eurpub/ckag062 (PMC13082903; doi:10.1093/eurpub/ckag062)
Supplement: ckag062_Supplementary_Data [file ckag062_supplementary_data.docx]

**Supplementary material**

***Data source***

The Longitudinal Integrated Database for Labour Market Studies (LISA) kept by Statistic Sweden (1), was used to obtain sociodemographic variables and annual information on income from work, parental leave, social assistance, unemployment, and student benefits, as well as old-age pension and emigration.

The following three registers held by the National Board of Health and Welfare, were used: (1) The National Patient Register (NPR) for information on main and side diagnoses with inpatient and specialized outpatient healthcare use, based on the 10^th^ version of the International Classification of Diseases (2); the Prescribed Drug Register for information on dispensed prescribed medications using the Anatomical-Therapeutic-Chemical [ATC] classification codes (3), and the Cause of Death Register. From the Social Insurance Agency, we used information on all annual days with net disability pension (DP) and sickness absence (SA) (in SA spells >14 days) from the Micro-Data for Analysis of the Social Insurance System (MiDAS) (6).

***Social insurance context in Sweden***

In Sweden, all residents aged 19-65 whose work capacity is permanently reduced due to disease or injury can be granted DP. All residents with income from work, parental leave benefits, or unemployment benefits with morbidity leading to work incapacity are eligible for SA. Both DP and SA can be granted for full- or part time (100%, 75%, 50%, or 25% of ordinary working hours), and it is possible to be on part-time SA and DP at the same time. There is no maximum duration of a SA-spell before age 65. SA benefits replace about 80% and DP about 64% of lost income, up to a certain limit. To account for part-time SA/DP in our study, the number of SA and DP net days was calculated by combining partial SA or DP gross days, e.g., a day with 75% SA and another day with 25% DP were counted as one net day of 100% SA/DP.

Sweden has no fixed retirement age, although there has been a strong social norm to retire at age 65 (7). For many years, including the here studied years, the minimum age to take out public pension was 61 years. This threshold was raised to 62 in 2020 (8), to 63 in 2023 (9) and will be raised to 64 in 2026 (9). Most employees can also receive employer-paid occupational pensions that are governed by collective agreements (10). Residents with no or minimum income can receive so called ‘guarantee pension’ from age 65 (this age limit was raised to 66 in 2023 and will be raised to 67 in 2026) (9).

***Definition of covariates***

Sociodemographic variables: country of birth (Sweden / other Nordic countries / Other European Countries / Rest of the word, educational level (primary school [≤9 years], including missing information (n=96) / high school [10–12 years] / university/college [>12 years]), type of living area (11) (big city / medium-sized city / small town/rural, and living arrangement (married or cohabiting / single). Family situation was first categorized as following: “married/cohabitant, without children below age 18 living at home”; “married/cohabitant, with children living at home”, “single, without children living at home”, “single, with children living at home”. However, as only a few lived with children younger than 18 years, and the numbers were especially low in some of these categories, we collapsed this variable to “married/cohabiting” vs. “single”.

Having had secondary healthcare (i.e., inpatient or specialized outpatient healthcare (using both main and side diagnoses (ICD-10 codes)) or prescribed medication) was used to identify people with specific morbidities, as described in the following.

*Mental disorders:* *common mental disorder* (depression: F32, F33; anxiety and stress-related disorders F40-43, Z73.0); *severe mental disorders* (psychosis spectrum: F20-29, bipolar disorder: F30, F31, early-onset neurodegenerative disorders: F00-F03, G30-G32)

*Common somatic diseases* (malignant neoplasm: C00-C97, diabetes: E10-E14, diseases of the circulatory system: I00-I99; asthma: J45, J46, musculoskeletal disorders M00-M99).

The following ATC codes prescribed medication use with a minimum of two dispersion within a year: psychopharmaceutical medication: N05A, N05B, N05C, N06A, N06B, N06C, N07B, and antidiabetics: A10.

***References***

1. Ludvigsson JF, Svedberg P, Olén O, Bruze G, Neovius M. The longitudinal integrated database for health insurance and labour market studies (LISA) and its use in medical research. Eur J Epidemiol. 2019 Apr;34(4):423–37.

2. Ludvigsson JF, Almqvist C, Bonamy AKE, Ljung R, Michaëlsson K, Neovius M, et al. Registers of the Swedish total population and their use in medical research. Eur J Epidemiol. 2016 Feb;31(2):125–36.

3. The ICD-10. International Statistical Classification of Diseases and Related Health Problems 10th Revision, Volume 2, Instruction Manual, 2010 Edition. Geneva: World Health Organization; 2011.

4. Wettermark B, Hammar N, Fored CM, Leimanis A, Otterblad Olausson P, Bergman U, et al. The new Swedish Prescribed Drug Register--opportunities for pharmacoepidemiological research and experience from the first six months. Pharmacoepidemiol Drug Saf. 2007 Jul;16(7):726–35.

5. World Health Organization [Internet]. Anatomical Therapeutic Chemical (ATC) Classification.

6. Österlund Niklas. MiDAS - sjukpenning och rehabiliteringspenning (MiDAS - sickness benefit and rehabilitation allowance)(in Swedish). Swedish Social Insurance Agency.; 2011.

7. The Swedish Social Insurance Agency. Förlängt arbetsliv - förutsättningar, utmaningar och konsekvenser: Rapport från forskarseminariet i Umeå 15–16 januari 2020. [Extended Working Life - Prerequisites, Challenges, and Consequences: Report from the Research Seminar in Umeå, January 15-16, 2020] (In Swedish). [Internet]. Umeå; 2020 [cited 2014 Oct 27]. Available from: www.forsakringskassan.se/download/18.7fc616c01814e179a9f32d/1656661298206/forlangt-arbetsliv-forutsattningar-utmaningar-och-konsekvenser-socialforsakringsrapport-2020-5.pdf

8. König S, Nerman M. Höjda åldersgränser i pensionssystemet [Raised age limits in the pension system]. Gothenbourg: Inspektionen för socialförsäkringen [Swedish Social Insurance Inspectorate]. Göteborg; 2023.

9. MinPension.se. Olika åldersgränser för pensionsuttag [Different age limits for pension withdrawals] 2024 [cited 2024 4 Oct]. [Internet]. 2024 [cited 2024 Oct 27]. Available from: https://www.minpension.se/allt-om-pensioner/ta-ut-pension/olika-aldersgranser-for-pensionsuttag

10.The Swedish pension system and pension protections until 2070. European Union; 2020.

11.Applying the Degree of Urbanisation – A methodological manual to define cities, towns and rural areas for international comparisons. European Union/FAO/UN-Habitat/OECD/The World Bank, 2021. doi: 10.2785/706535.

**Supplementary Tables**

**Supplementary Table 1**
Median income in Sweden during the study period, by Statistics Sweden (www.scb.se^*^)

| Year | Monthly median income (in Swedish kronor) | Yearly price base amount (in Swedish kronor) |
| --- | --- | --- |
| 2000 | 18,100 | 36,600 |
| 2001 | 19,000 | 36,900 |
| 2002 | 19,700 | 37,900 |
| 2003 | 20,300 | 38,600 |
| 2004 | 21,000 | 39,300 |
| 2005 | 21,600 | 39,400 |
| 2006 | 22,200 | 39,700 |
| 2007 | 23,000 | 40,300 |
| 2008 | 24,000 | 41,000 |
| 2009 | 24,900 | 42,800 |
| 2010 | 25,300 | 42,400 |
| 2011 | 25,900 | 42,800 |
| 2012 | 26,600 | 44,000 |
| 2013 | 27,300 | 44,500 |
| 2014 | 28,000 | 44,400 |
| 2015 | 28,600 | 44,500 |
| 2016 | 29,300 | 44,300 |
| 2917 | 30,000 | 44,800 |
| 2018 | 30,900 | 45,500 |
| 2019 | 31,700 | 46,500 |

*Note.* The same thresholds were used for women and men.

*https://www.scb.se/hitta-statistik/sverige-i-siffror/utbildning-jobb-och-pengar/medianloner-i-sverige/

**Supplementary Table 2a**
Transition probabilities between different activities in women

| **Origin state** | **Transiting state** | | | | | | |
| --- | --- | --- | --- | --- | --- | --- | --- |
|  | *High income* | *Low income* | *No/minimal income* | *On social assistance* | *Retired* | *SA/DP* | *Unemployed* |
| *High income* | 0.863 | 0.104 | 0.001 | <0.001 | 0.017 | 0.009 | 0.005 |
| *Low income* | 0.062 | 0.825 | 0.007 | <0.001 | 0.055 | 0.036 | 0.015 |
| *No/minimal income* | 0.007 | 0.125 | 0.669 | 0.020 | 0.105 | 0.034 | 0.040 |
| *On social*  *assistance* | <0.001 | 0.028 | 0.046 | 0.800 | 0.034 | 0.062 | 0.030 |
| *Retired* | 0.001 | 0.012 | 0.002 | 0.001 | 0.979 | 0.003 | 0.001 |
| *SA/DP* | 0.008 | 0.083 | 0.008 | 0.003 | 0.060 | 0.816 | 0.023 |
| *Unemployed* | 0.016 | 0.168 | 0.026 | 0.005 | 0.065 | 0.085 | 0.636 |

*Note.* SA/DP: sickness absence and/or disability pension

**Supplementary Table 2b**

Transition probabilities between different activities in men

| Origin state | Transiting state | | | | | | |
| --- | --- | --- | --- | --- | --- | --- | --- |
|  | *High income* | *Low income* | *No/minimal income* | *On social assistance* | *Retired* | *SA/DP* | *Unemployed* |
| *High income* | 0.882 | 0.080 | 0.002 | <0.001 | 0.021 | 0.007 | 0.008 |
| *Low income* | 0.119 | 0.734 | 0.018 | 0.001 | 0.072 | 0.027 | 0.029 |
| *No/minimal income* | 0.023 | 0.173 | 0.618 | 0.021 | 0.096 | 0.025 | 0.043 |
| *On social assistance* | 0.001 | 0.041 | 0.045 | 0.742 | 0.041 | 0.064 | 0.067 |
| *Retired* | 0.005 | 0.019 | 0.003 | 0.001 | 0.969 | 0.003 | 0.001 |
| *SA/DP* | 0.016 | 0.059 | 0.010 | 0.004 | 0.065 | 0.815 | 0.030 |
| *Unemployed* | 0.033 | 0.141 | 0.022 | 0.009 | 0.069 | 0.050 | 0.676 |

*Note.* SA/DP: sickness absence and/or disability pension

**Supplementary Table 3a**
Measures of cluster partitions quality for different numbers of clusters in women

| **Number of clusters** | **PBC** | **HG** | **HGSD** | **ASW** | **ASWw** | **CH** | **R2** | **CHsq** | **R2sq** | **HC** |
| --- | --- | --- | --- | --- | --- | --- | --- | --- | --- | --- |
| 2 | 0.53 | 0.85 | 0.85 | 0.67 | 0.67 | 12.423 | 0.19 | 18.917 | 0.26 | 0.20 |
| 3 | 0.51 | 0.89 | 0.89 | 0.56 | 0.56 | 12.828 | 0.33 | 15.153 | 0.36 | 0.10 |
| 4 | 0.58 | 0.94 | 0.94 | 0.60 | 0.60 | 13.735 | 0.44 | 30.398 | 0.63 | 0.03 |
| 5 | 0.43 | 0.83 | 0.83 | 0.41 | 0.41 | 12.847 | 0.49 | 24.202 | 0.65 | 0.06 |
| 6 | 0.31 | 0.77 | 0.77 | 0.41 | 0.41 | 12.112 | 0.53 | 20.089 | 0.65 | 0.06 |
| 7 | 0.32 | 0.82 | 0.82 | 0.44 | 0.44 | 11.721 | 0.57 | 19.139 | 0.68 | 0.04 |

PBC: Point Biserial Correlation

HG: Hubert’s Gamma

HGSD: Hubert’s Somers’ D

ASW: Average Silhouette Width

ASWw: Average Silhouette Width (weighted)

CH: Calinski-Harabasz index

R2: Pseudo R²

CHsq: Calinski-Harabasz index squared

R2sq: Pseudo R² squared

HC: Hubert’s C

**Supplementary Table 3b**
Measures of cluster partitions quality for different numbers of clusters in men

| **Numer of clusters** | **PBC** | **HG** | **HGSD** | **ASW** | **ASWw** | **CH** | **R2** | **CHsq** | **R2sq** | **HC** |
| --- | --- | --- | --- | --- | --- | --- | --- | --- | --- | --- |
| 2 | 0.53 | 0.85 | 0.85 | 0.65 | 0.65 | 12.455 | 0.19 | 18.577 | 0.26 | 0.20 |
| 3 | 0.67 | 0.92 | 0.92 | 0.65 | 0.65 | 12.369 | 0.32 | 34.436 | 0.57 | 0.06 |
| 4 | 0.53 | 0.88 | 0.88 | 0.50 | 0.50 | 12.736 | 0.42 | 29.020 | 0.63 | 0.05 |
| 5 | 0.39 | 0.74 | 0.74 | 0.35 | 0.35 | 11.587 | 0.47 | 22.939 | 0.64 | 0.07 |
| 6 | 0.40 | 0.76 | 0.76 | 0.38 | 0.38 | 10.829 | 0.51 | 22.570 | 0.69 | 0.06 |
| 7 | 0.34 | 0.79 | 0.79 | 0.40 | 0.40 | 10.372 | 0.55 | 19.435 | 0.69 | 0.05 |

PBC: Point Biserial Correlation

HG: Hubert’s Gamma

HGSD: Hubert’s Somers’ D

ASW: Average Silhouette Width

ASWw: Average Silhouette Width (weighted)

CH: Calinski-Harabasz index

R2: Pseudo R²

CHsq: Calinski-Harabasz index squared

R2sq: Pseudo R² squared

HC: Hubert’s C

**Supplementary Figures**

**Supplementary Figure 1.** Algorithm for the definition of yearly mutually exclusive labor market states

**
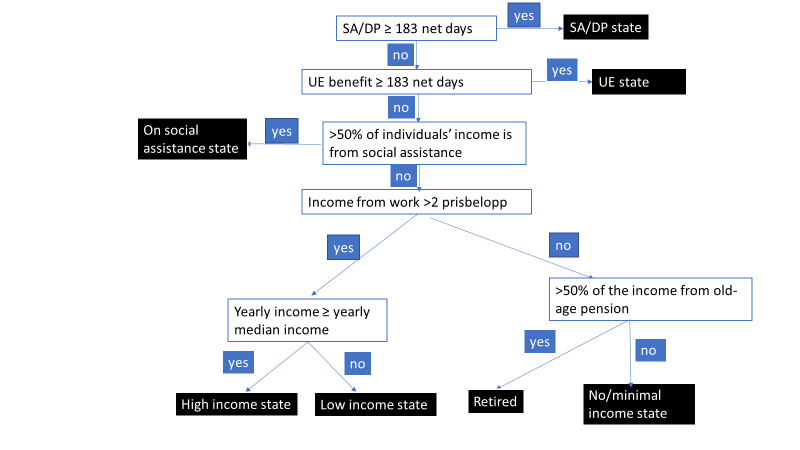
**

*Note.* SA/DP = sickness absence and/or disability pension. UE = unemployment. Prisbelopp = price base amount.

**Supplementary Figure 2.** 20-year retrospective sequence index plots (sorted by state at age 47), sequnce distribution plot and 10 most common sequences for **women** and **men** aged 66 years in 2019


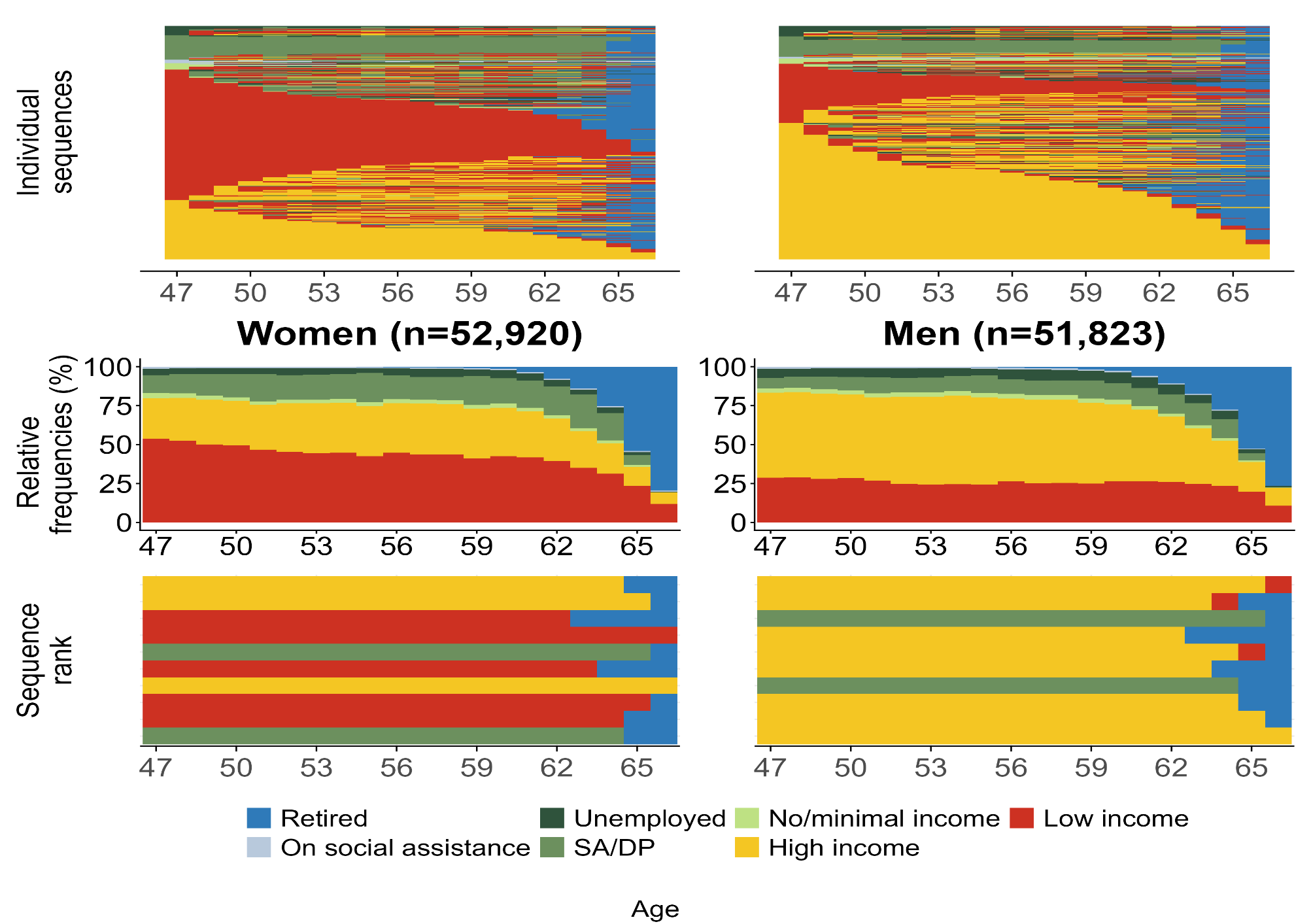


*Note.* SA/DP = sickness absence/disability pension

**Supplementary Figure 3.** 20-year retrospective sequence index plots sorte by age 66 for women and men.


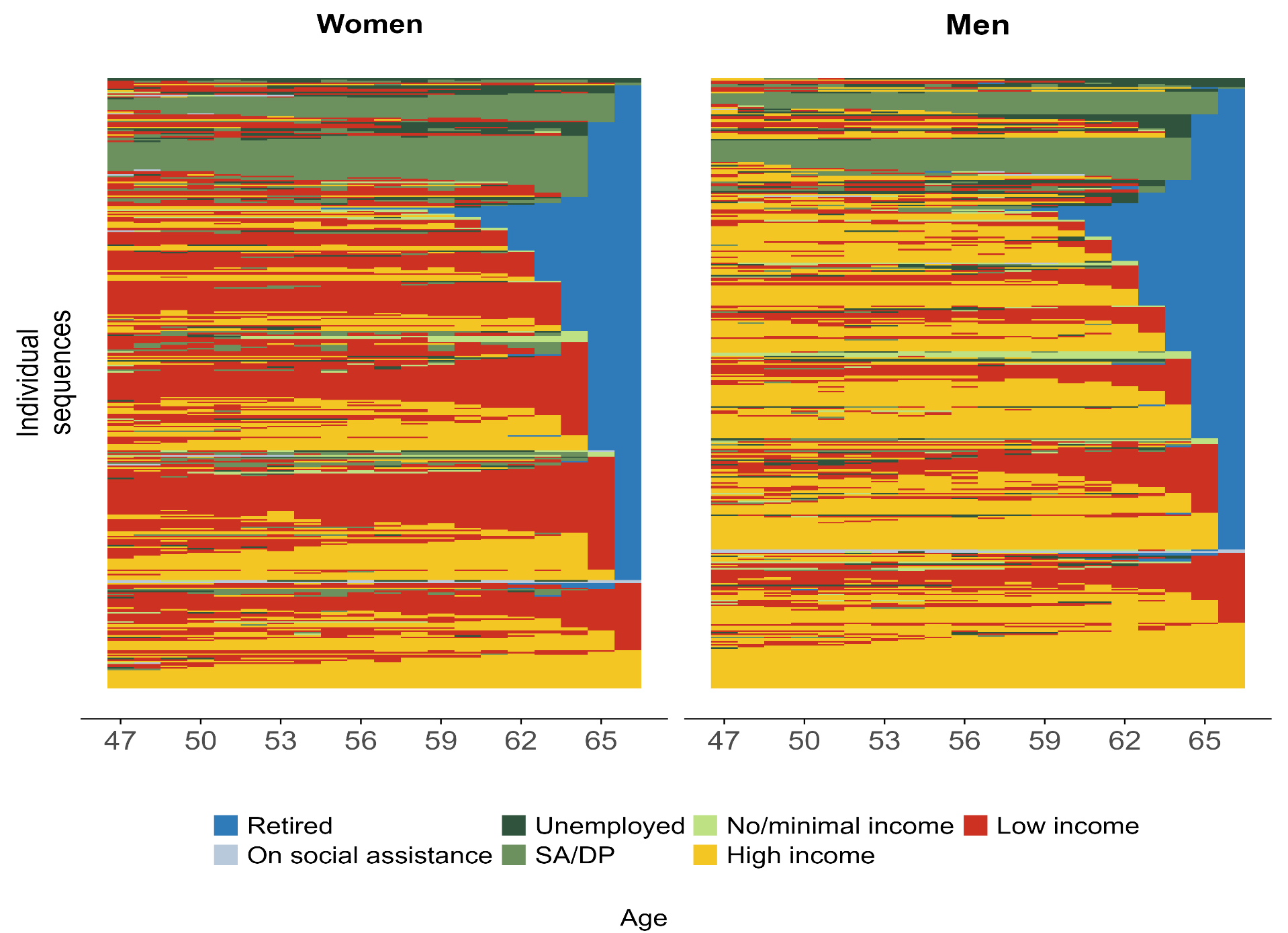


*Note.* SA/DP = sickness absence/disability pension


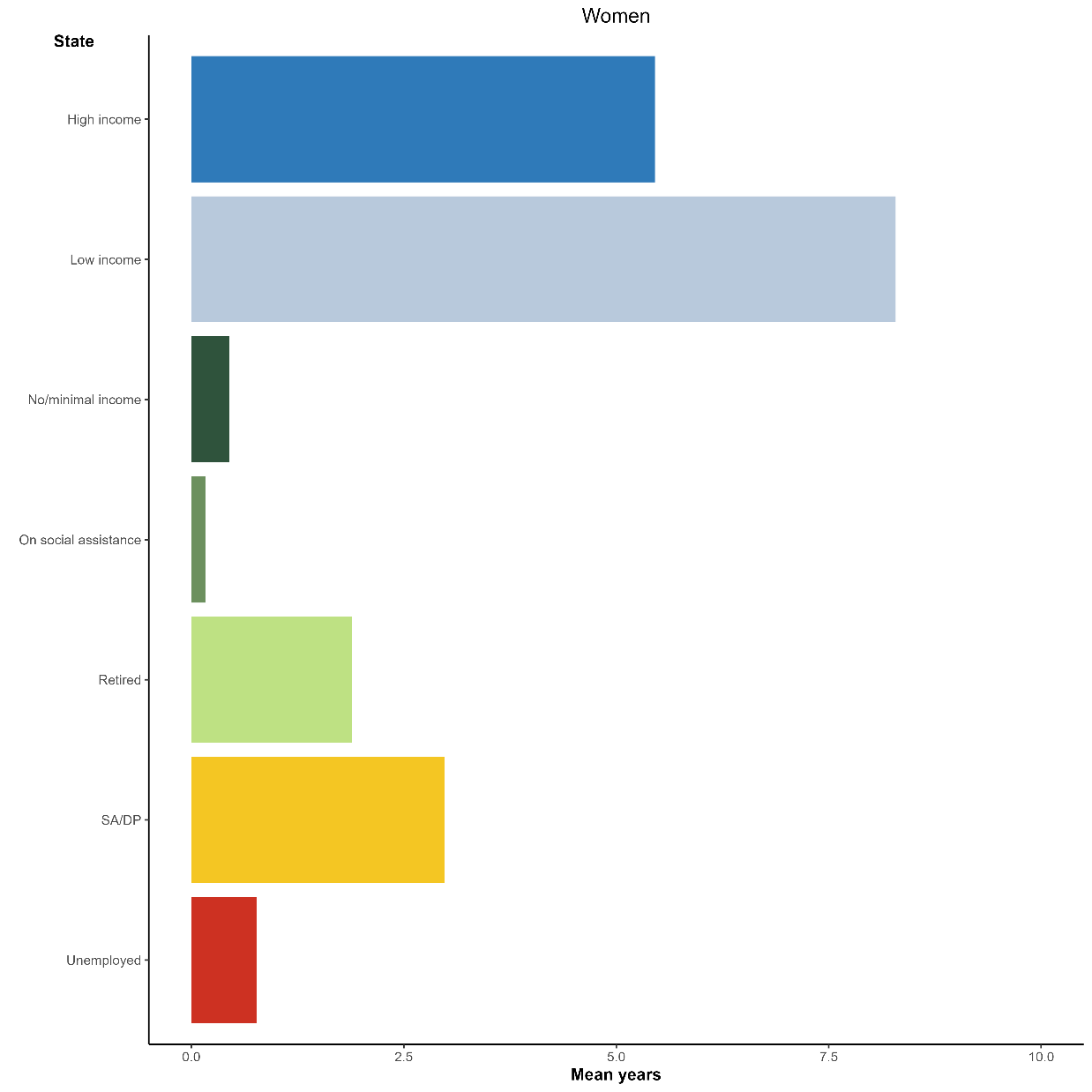

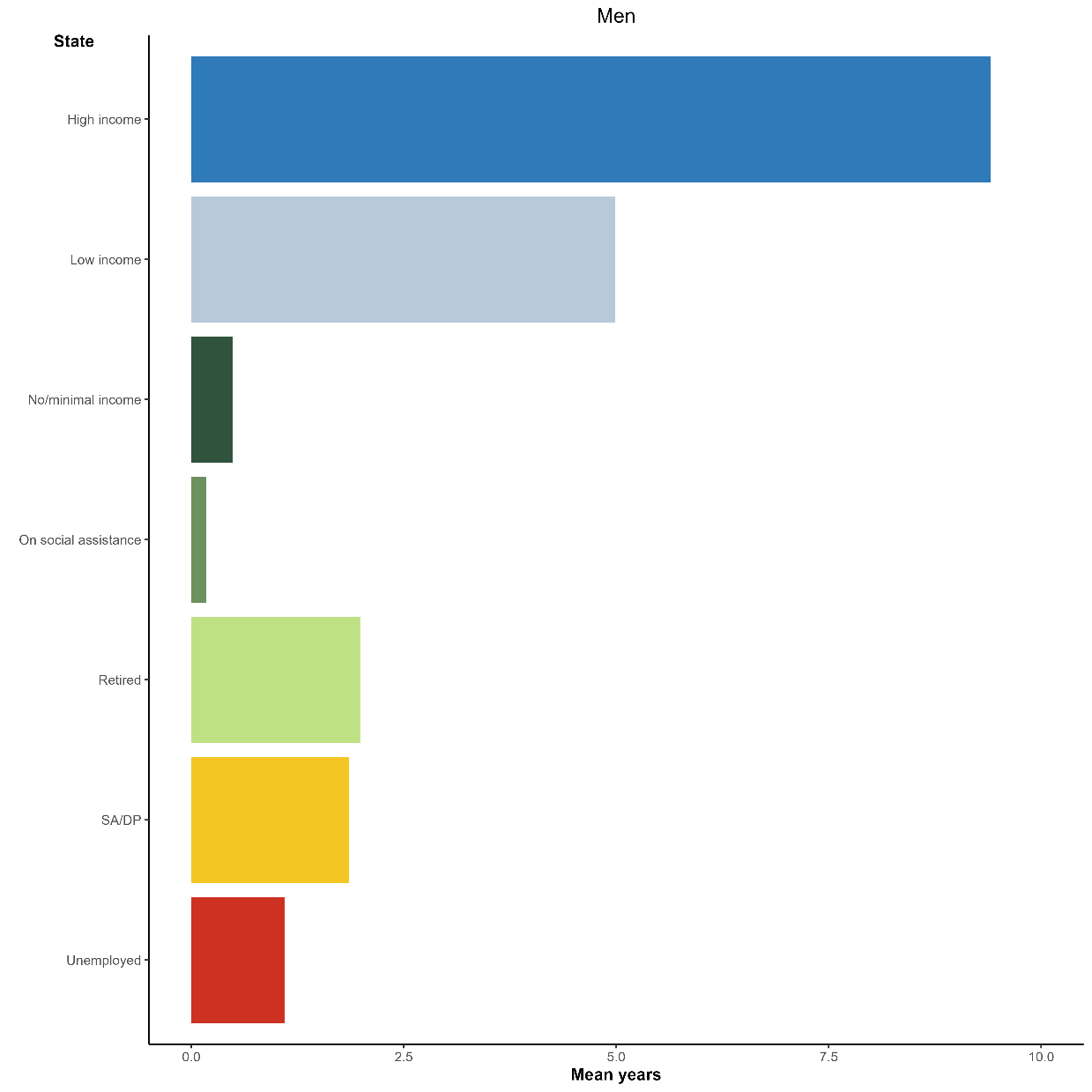
**Supplementary Figure 4.** Mean time spent in different labor market states in women (left) and in men (right)

*Note*. SA/DP = sickness absence/disability pension

**Supplement Figure 5a.** Sequence index plot of 400 randomly selected individual sequences by cluster, sorted by age 66 for women


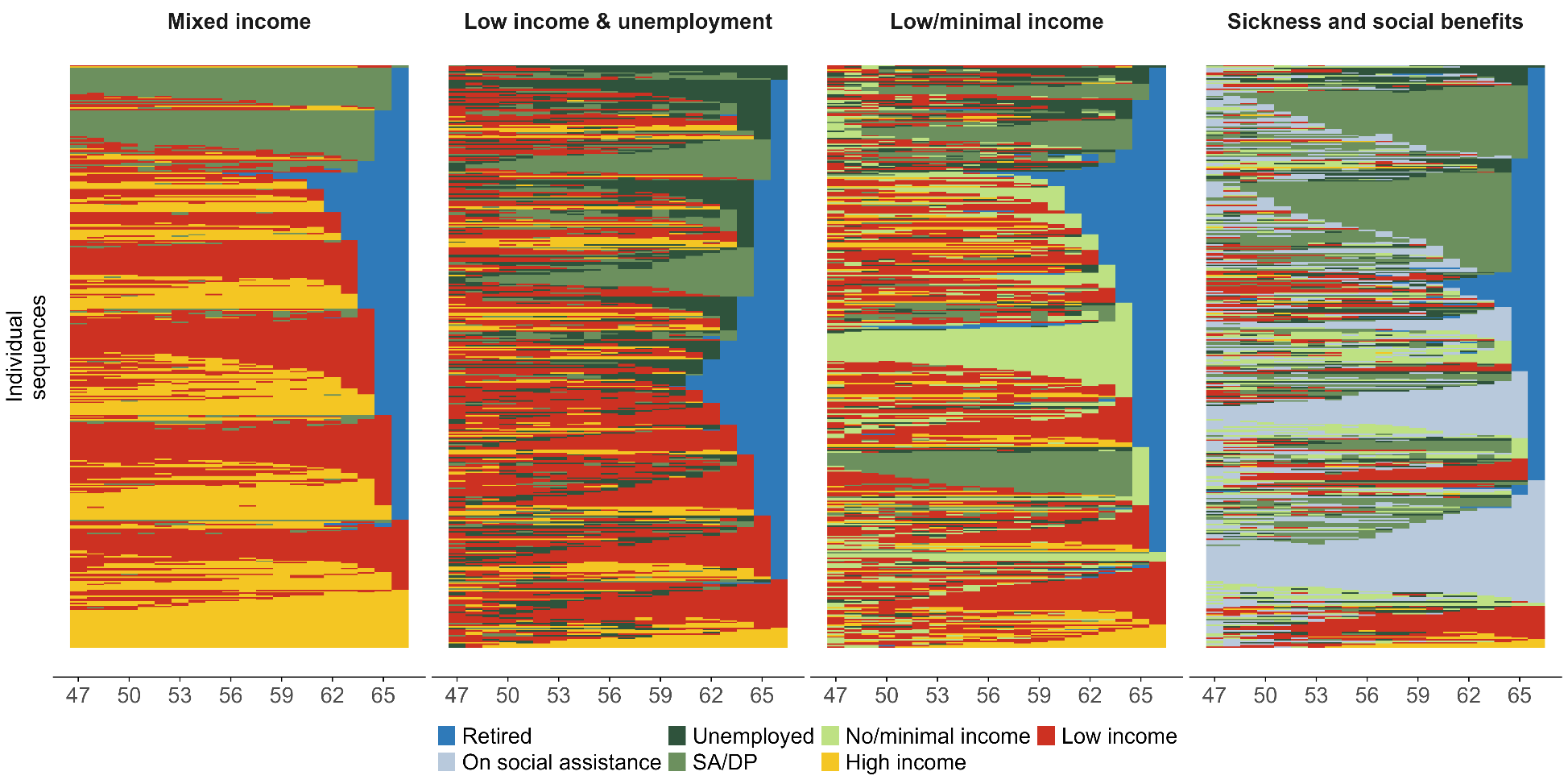


*Note.* SA/DP = sickness absence/disability pension

**Supplement Figure 5b.** Sequence index plot of 400 randomly selected individual sequences by cluster, sorted by age 66 for men


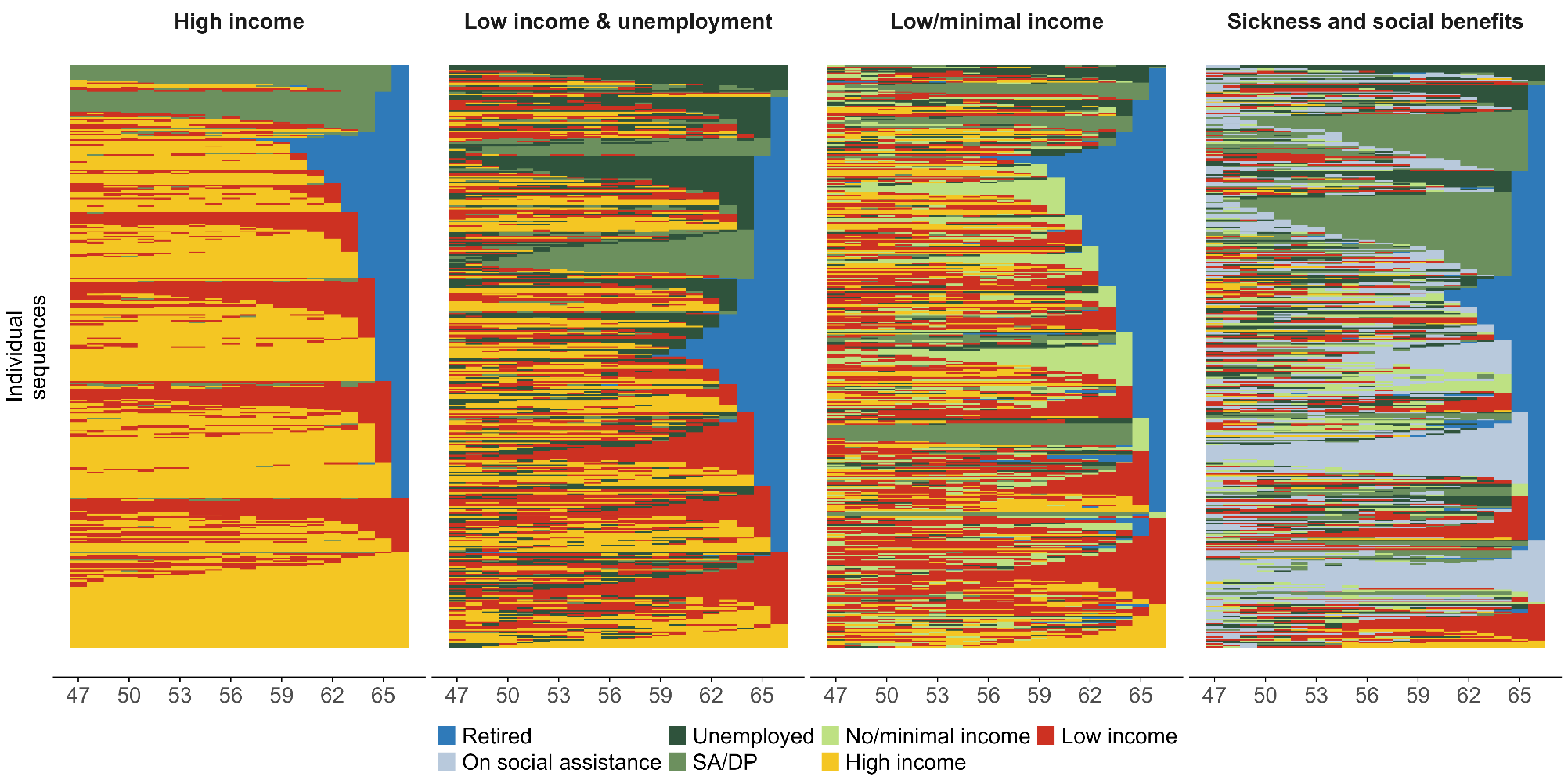


*Note.* SA/DP = sickness absence/disability pension

**Supplementary Figure 6a.** Sequence dissection figure for women’s working life clusters


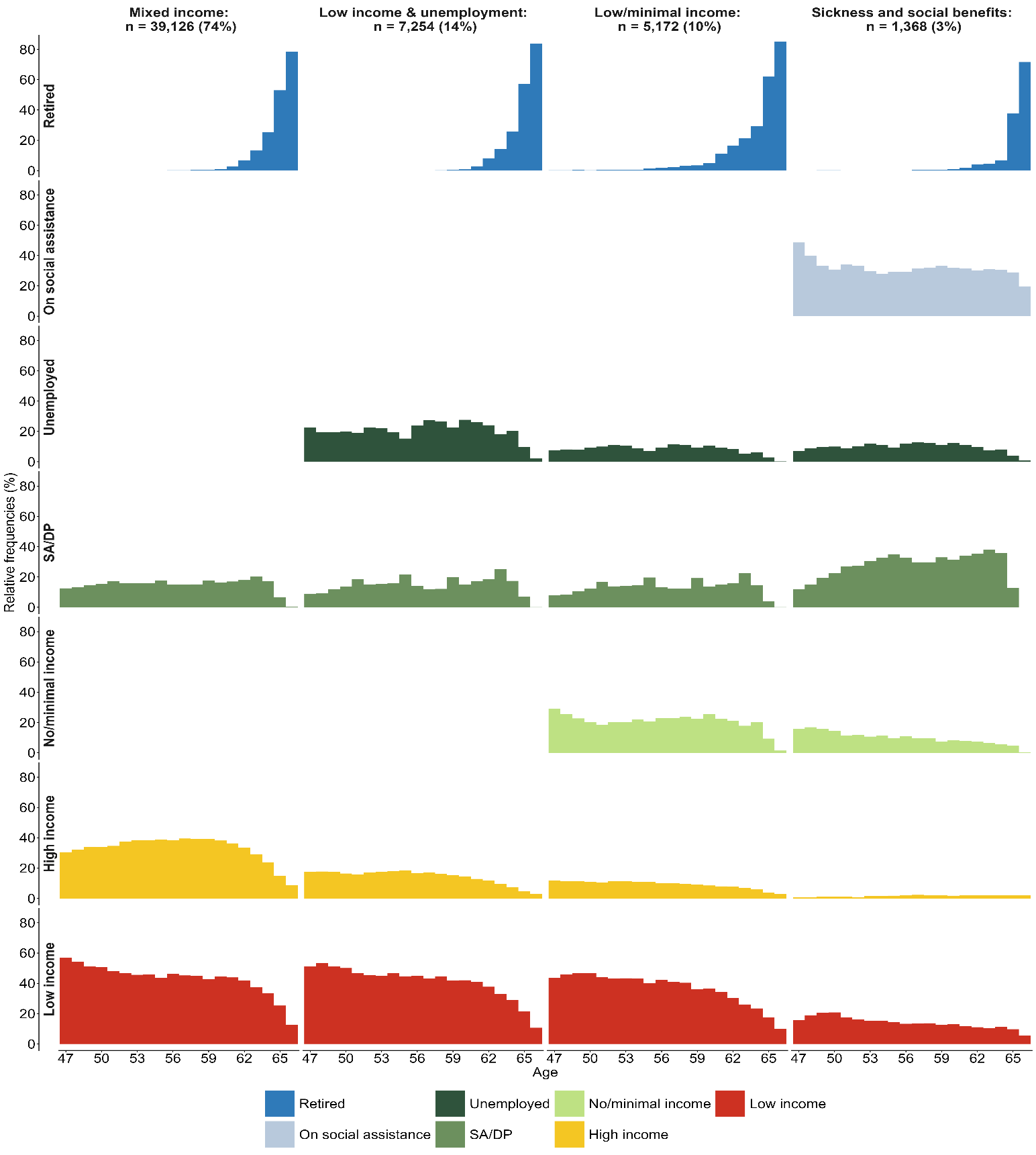


*Note.* SA/DP = sickness absence/disability pension

**Supplementary Figure 6b.** Sequence dissection figure for men’s working life clusters


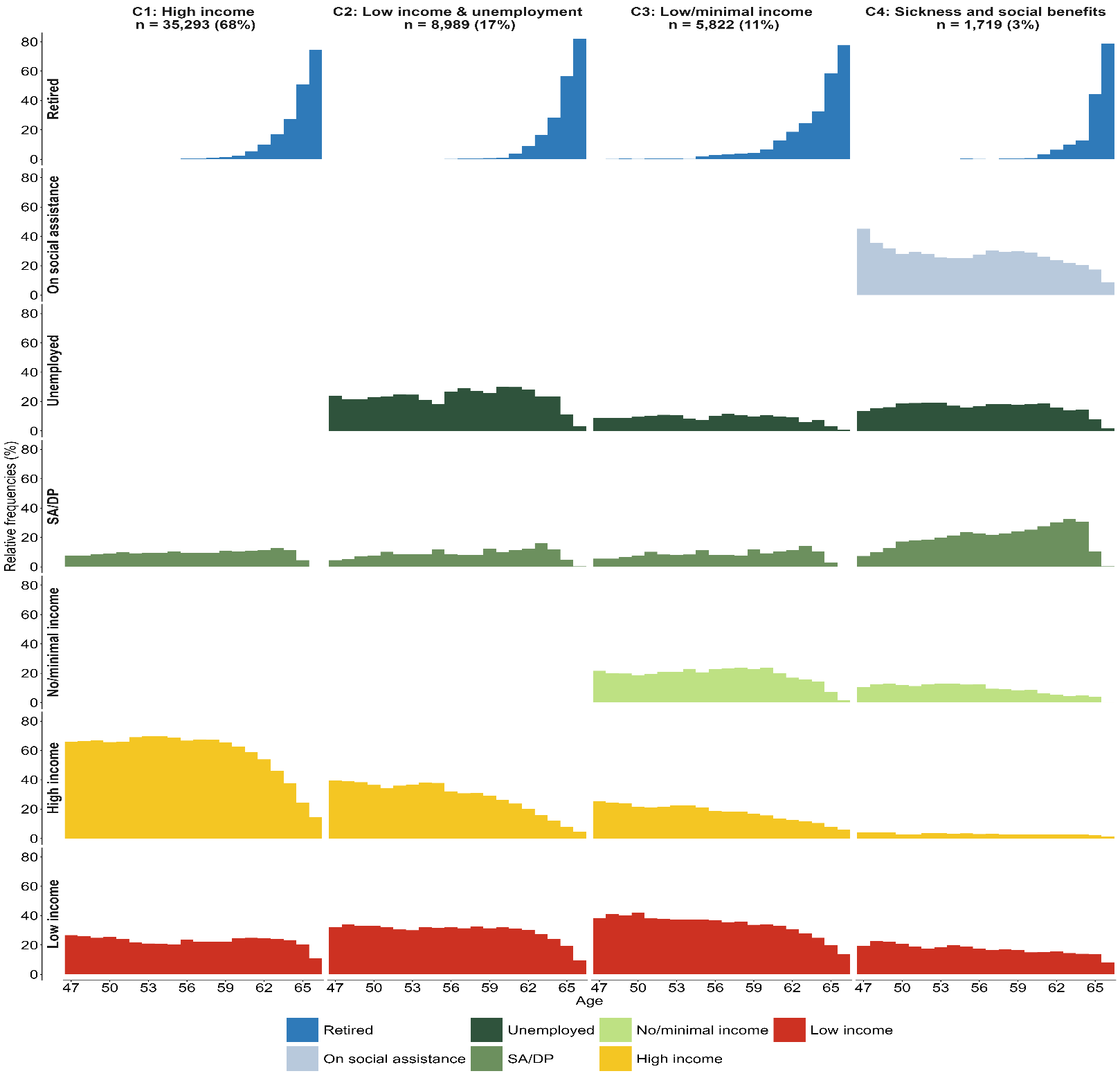


*Note.* SA/DP = sickness absence/disability pension

**Supplementary Figure 7a.** Alternative, two-cluster solution for women


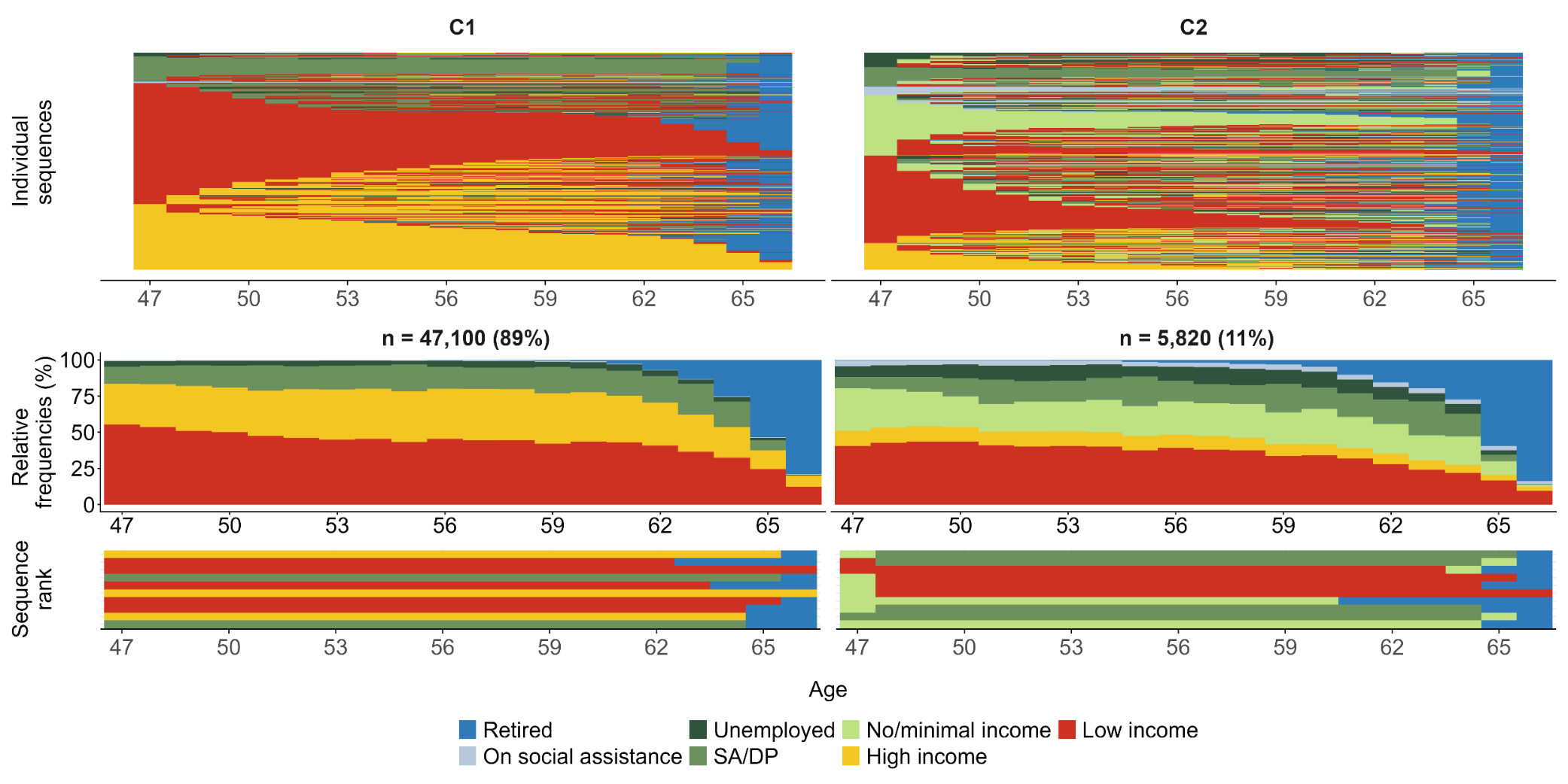


*Note.* C1 = Cluster 1, C2 = Cluster 2; SA/DP = sickness absence/disability pension. The upper panel shows a random sample of 400 individual sequences (number of sequences is limited due to resolution constraints), sorted by the state at age 47. The sequence rank plot in the lower panel shows the ten most common sequences within each cluster.

**Supplementary Figure 7b.** Alternative, two-cluster solution for women


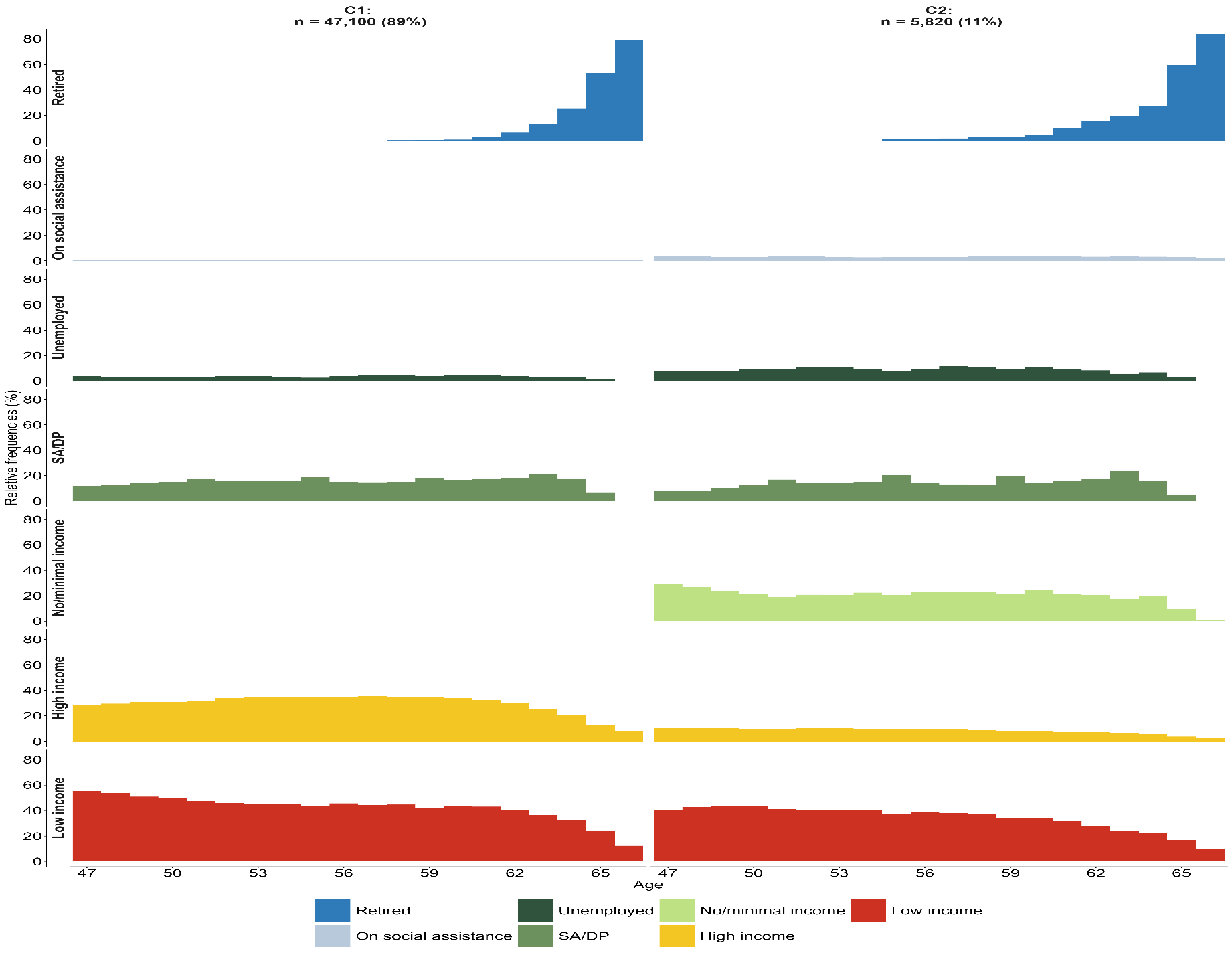


*Note.* C1 = Cluster 1, C2 = Cluster 2, SA/DP = sickness absence/disability pension.

**Supplementary Figure 8a.** Alternative, three-cluster solution for men


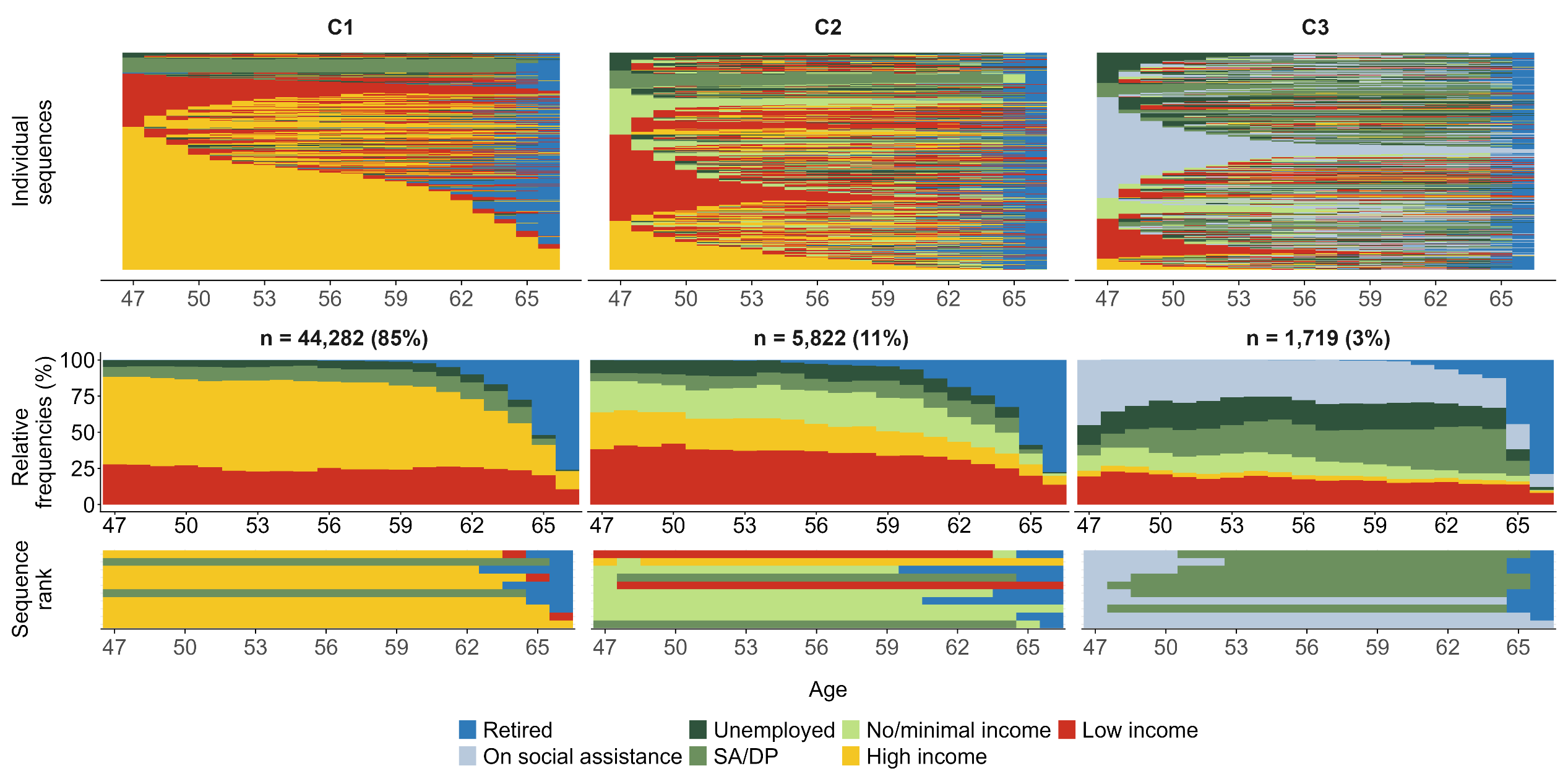


*Note.* C1 = Cluster 1, C2 = Cluster 2, SA/DP = sickness absence/disability pension. The upper panel shows a random sample of 400 individual sequences (number of sequences is limited due to resolution constraints), sorted by the state at age 47. The sequence rank plot in the lower panel shows the ten most common sequences within each cluster.

**Supplementary Figure 8b.** Alternative, three-cluster solution for men, decomposed version


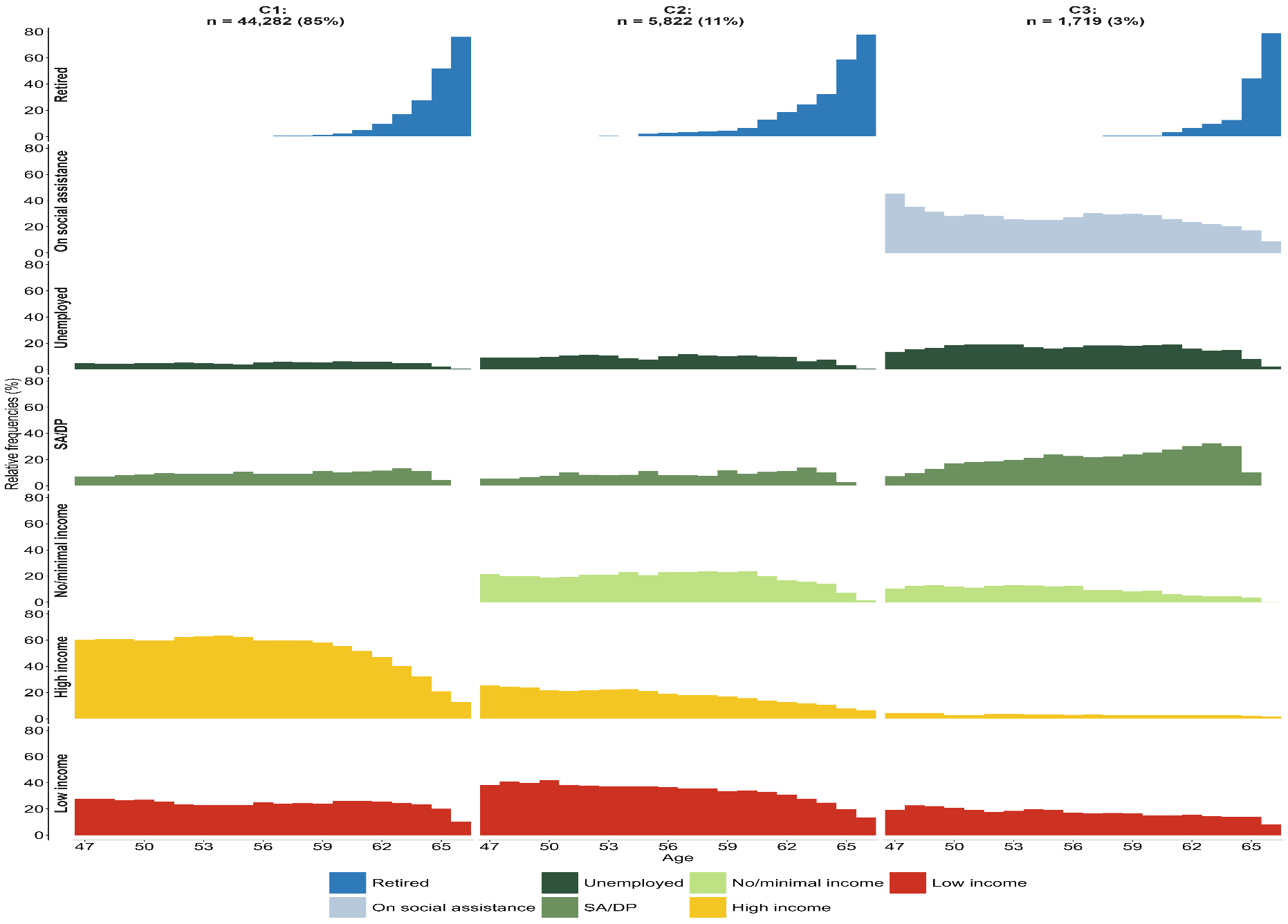


*Note.* C1 = Cluster 1, C2 = Cluster 2, C3 = cluster 3, SA/DP = sickness absence/disability pension.
